# Improvement of Recalcitrant Dissecting Cellulitis of the Scalp After a Trial of Upadacitinib

**DOI:** 10.7759/cureus.52377

**Published:** 2024-01-16

**Authors:** Zahidul Islam, Michelle Toker, Isha M Gandhi, Ariel Sher, Kristina Campton

**Affiliations:** 1 Division of Dermatology, Albert Einstein College of Medicine, Bronx, USA; 2 Dermatology, University of Minnesota Twin Cities Medical School, Minneapolis, USA; 3 Dermatology, New York Medical College, Valhalla, USA

**Keywords:** hidradenitis suppurativa, follicular occlusion tetrad, upadacitinib, jak inhibitor, folliculitis decalvans, dissecting cellulitis of scalp

## Abstract

Dissecting cellulitis of the scalp (DCS) is a rare condition characterized by painful inflammatory nodules and abscesses on the scalp, often leading to sinus tracts and scarring alopecia. We present a case of DCS in a 26-year-old male who experienced significant clinical improvement following a short course of upadacitinib, a Janus kinase (JAK) inhibitor. The patient received multiple standard treatments such as topical antimicrobials, oral antibiotics, corticosteroids, and intralesional triamcinolone injections, with limited success. However, following the initiation of upadacitinib, the patient reported reduced pain, pustular draining, and bleeding, with significantly improved quality of life. To our knowledge, there is currently a paucity of literature documenting the use of JAK inhibitors for DCS. This case aims to highlight the potential of JAK inhibitors as a therapy for refractory DCS, a condition with limited treatment options.

## Introduction

Dissecting cellulitis of the scalp (DCS) is a rare condition that is a part of the follicular occlusion tetrad of hidradenitis suppurativa (HS), acne conglobata, and pilonidal disease [1]. It commonly occurs in 20-40-year-old African American males and presents as painful, inflammatory nodules or abscesses that can precipitate the development of sinus tracts, keloids, and scarring alopecia [2]. Although the precise etiology and inflammatory cytokines involved in DCS are unclear, it is suspected that an error in follicular keratinization leads to the obstruction and rupture of hair follicles, resulting in a chronic inflammatory response [1]. HS is a condition closely linked with DCS and thus, one of the pathways that could possibly be involved in the chronic inflammatory response is the Janus kinase (JAK) signaling pathway [3]. JAK inhibitors bind to JAK, an intracellular tyrosine-kinase protein, which downregulates pro-inflammatory signaling molecules such as IL-10, IL-12, and IL-23 [4]. Upadacitinib is highly selective for the JAK1 isoform and inhibits IL-6 and interferon gamma-related signaling [5]. We present a case of recalcitrant DCS that displayed an impressive clinical response after a course of upadacitinib.

## Case presentation

**Figure 1:**
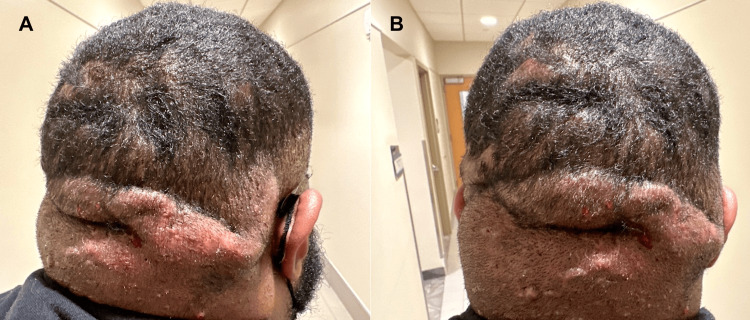
Prior to the trial of upadacitinib Posterior scalp with large pustules, nodules, and interconnected, firm, edematous sinus tracts that drain pus and blood, with an absence of hair growth.

The patient is a 26-year-old male, non-smoker with a history of obesity and atopic dermatitis. On his initial visit, he reported an 11-month history of enlarging, painful cysts on the posterior scalp that drained purulent exudate and blood. He had not experienced similar cutaneous symptoms at other sites, including the axillae or groin, and never had prior treatment for his scalp lesions. On physical examination, the posterior scalp exhibited large, tender, draining pustules, nodules, and interconnected, firm sinus tracts with absent hair growth (Figure 1A, 1B). A complete blood count and basic metabolic panel were within normal limits. Inflammatory markers, including C-reactive protein (1.4 mg/dL, ref <0.8), erythrocyte sedimentation rate (60 mm/h, ref 0-15), and IL-6 (5.73 pg/mL, ref <5.0), were elevated.

**Figure 2:**
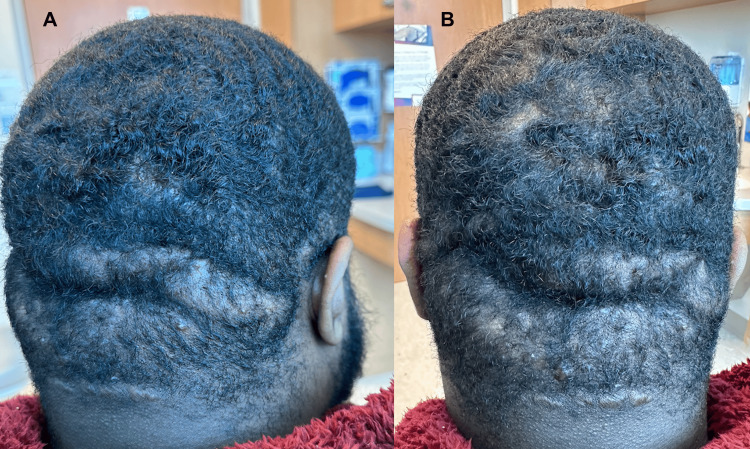
After two months of upadacitinib Posterior scalp with fewer pustules and smaller, less inflamed sinus tracts without pustular drainage or bleeding. Areas of prior inflammation now exhibit scarring.

Prior to therapy with oral JAK inhibitor upadacitinib, the patient was given topical antimicrobials (benzoyl peroxide 10%), oral antibiotics (sulfamethoxazole-trimethoprim 800-160 mg twice daily), and oral prednisone (20 mg three times a day). Intralesional triamcinolone injections (40 mg/ml) were also administered to the affected areas over multiple follow-up visits. Adalimumab could not be ordered due to insurance and financial issues. Undergoing surgery or taking isotretinoin was also not preferred by the patient. Four months following his initial visit, the patient stated that only short-term relief was felt with the corticosteroid injections and there was little to no improvement in his overall condition since beginning treatment. A physical examination revealed continued tenderness, inflammation, and drainage from his scalp lesions. Due to limited therapies available for the treatment of DCS and the failure of standard therapies, a trial of upadacitinib 15 mg twice daily was initiated and added to his regimen of topical antimicrobials, oral antibiotics, and corticosteroid injections. At a one-month follow-up visit, the patient reported substantial improvement in pain, pustular draining, and bleeding. According to the patient, this was the longest period where he experienced minimal to no flares and reported a dramatic improvement in his quality of life. At around a two-month follow-up visit, a physical exam revealed significantly fewer pustules, smaller sinus tracts, and overall decreased inflammation with no visible drainage (Figure 2A, 2B). No major side effects were reported and the continuation of upadacitinib was planned.

## Discussion

Features of DCS overlap with many core qualities of HS, such as the type of lesions present, recurrence of lesions, and its suppurative nature, with the main difference being the location of the disease. These two diseases often coexist [1]. Oral JAK1 inhibitors have shown preliminary efficacy in treating moderate-to-severe HS in two phase II studies, have demonstrated high clinical responses in cohort studies, and are currently undergoing a phase III trial for HS [3,6]. However, it is currently only FDA-approved for varying inflammatory and rheumatologic conditions such as rheumatoid arthritis, psoriatic arthritis, atopic dermatitis, and recently for moderate-to-severe Crohn’s disease [7,8].

Current standard pharmacological treatments for DCS remain limited and include oral antibiotics, isotretinoin, and corticosteroids, which all have variable efficacy [9]. In recent case studies, tumor necrosis factor-alpha inhibitors have shown some promise in DCS refractory to conventional treatment, a therapy also indicated for moderate-to-severe HS [10,11]. One recent case of an adolescent with DCS demonstrated a successful response to treatment with adalimumab and baricitinib [1]. However, there is no literature detailing the use of JAK inhibitors as a potential therapy for DCS, and to our current knowledge, this is the first case of recalcitrant DCS successfully responding to a trial of upadacitinib. Although our patient was not taking upadacitinib as a monotherapy for his condition and was on other concomitant medications, it was only after the addition of upadacitinib to his regimen that a significant clinical response was observed after months of failing to respond to standard therapies.

## Conclusions

Our case adds to the increasing number of literature on the use of anti-inflammatory agents like JAK inhibitors for the treatment of inflammatory and suppurative dermatoses. This single-patient observation introduces the need for further investigation and consideration of including JAK inhibitors in the management of refractory DCS, a chronic, difficult-to-eradicate disease for which current treatment options are limited. 
